# Diurnal and geographic variations of pollinator importance for *Cucurbita maxima* Duchesne

**DOI:** 10.1002/ece3.10651

**Published:** 2023-10-31

**Authors:** Mito Ikemoto, Yoshihiro Tanaka, Katsuyuki Kohno, Tomoyuki Yokoi

**Affiliations:** ^1^ Faculty of Life and Environmental Sciences University of Tsukuba Tsukuba Japan; ^2^ National Institute for Environmental Studies Tsukuba Japan; ^3^ Kagoshima Prefectural Institute for Agricultural Development Minamisatsuma Japan; ^4^ Institute of Vegetable and Floriculture Science, National Agriculture and Food Research Organization Tsu Japan

**Keywords:** crop pollination, Cucurbitaceae, pollinator diversity, sustainable agriculture

## Abstract

Despite growing awareness of the importance of monitoring wild crop pollinators worldwide, there are still few reports, especially in East Asia. Considering ongoing global warming may change the distribution range and diurnal activity of pollinators, it is necessary to describe current geographic and diurnal patterns. We clarified pollinators of *Cucurbita maxima* Duchesne (Cucurbitales: Cucurbitaceae) in three geographically distinct (>350 km, minimum) areas in Japan, focusing on diurnal variation. *Apis mellifera* L. (Hymenoptera: Apidae) and Halictidae (Hymenoptera) were observed in all of the experimental gardens. *Apis cerana japonica* Radoszkowski (Hymenoptera: Apidae) were mainly observed in Mie and Kagoshima, while *Bombus diversus diversus* Smith (Hymenoptera: Apidae) were observed only in Ibaraki. The peak time of flower visits depended both on bee taxa and area, and interestingly, did not necessarily synchronize with the timing of the highest pollen loads and the probability of stigma contact. In particular, visits and probability of contacting stigmas of Halictidae tended to increase as time passed, whereas pollen grains on their bodies sharply decreased with time; only a few individuals of Halictidae that visit early can become effective pollinators. There were no differences in yields between supplementary hand and natural pollination in all areas, and flower‐enclosure experiments using different mesh sizes clarified that small insects that can go across an approximately 4‐mm mesh may not transport sufficient pollen for fruit set. Our study demonstrated that pollination effectiveness, which is usually regarded as a static value, within a taxon can fluctuate in the space of just several hours. Considering such diurnal patterns can be altered by climate change, we need to carefully monitor the diurnal temporal patterns of pollinators worldwide.

## Graphical abstract

In this study, we clarified flower‐visiting insects of *Cucurbita maxima* in three sites in distinct areas of Japan and assessed the relative importance of the insects as pollinators, considering diurnal variations. Notably, we clearly showed diurnal changes in the pollen loads of each insect group, despite that it was generally regarded as a constant within a day. We concluded that *Bombus diversus diversus* and two honeybee species strongly contributed to the pollination success in the study sites, whereas Halictidae weakly did.

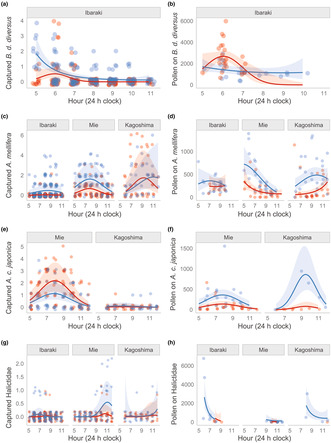

## 1 INTRODUCTION

Most flowering plants rely on animals for pollen transport (Ollerton et al., 2011), and crops are no exception, as it has been shown that 87 of 124 worldwide leading crops need animal pollination to some extent (Klein et al., 2007). Agricultural demand for animal pollination has been increasing since at least 1961 (Aizen et al., 2008), much faster than the supply of managed honeybees (Aizen & Harder, 2009). This implies the necessity of wild pollinators for crop production, but various wild bee populations are declining at a global scale (Aldercotte et al., 2022; Goulson et al., 2015). Therefore, monitoring flower‐visiting insects of individual crop species in diverse areas and clarifying those which act as pollinators is an urgent issue (Allen‐Perkins et al., 2022; Garibaldi et al., 2020). In this context, Allen‐Perkins et al. (2022) successfully developed a worldwide crop pollination database covering 48 commercial crop species from 34 countries. However, East Asia was limited to two crop species (*Fagopyrum esculentum* Moench and *Brassica napus* L.) of Japan and China in this database (Allen‐Perkins et al., 2022), implying a scarcity of studies in East Asia. This is despite East Asia being recognized as one of the highest pollinator dependent areas for crop production (Lautenbach et al., 2012).

Regarding evaluations of flower visitors as pollinators, it is widely accepted that pollinator importance of each taxonomic group is shaped by frequency of flower visits and pollination effectiveness (in a broad sense, degrees of contribution to pollen deposition to stigma or to seed set per visit) (Olsen, 1997; Schemske & Horvitz, 1984; Stebbins, 1970; Waser & Price, 1983 reviewed by Ne'Eman et al., 2010). Frequency of insect visits to each flower species often varies within a day, and many studies have pointed out temporal complementarity can occur between pollinator species visiting at different times (Albrecht et al., 2012; Fleming et al., 2001; Pisanty et al., 2016). On the other hand, pollination effectiveness of each species is often regarded as a static value within a day, and whether temporal variations exist has rarely been examined (but see Pfister et al., 2017). Yet, assessment of diurnal temporal variations of pollination effectiveness is essential; temperature often constrains pollen viability (e.g., Hayase, 1974b) and insect activity (e.g., Kenna et al., 2021), and thus ongoing global warming may potentially alter their patterns in the near future.

Squashes (*Cucurbita* spp., Cucurbitaceae, Cucurbitales) are a globally commercially important crop, and these diclinous plants need animal pollination for fruit and seed set. On the American continent, with native areas of squashes, specialist squash bees (*Eucera* spp.) act as pollinators, in addition to bumblebees (*Bombus* spp.) and *Apis mellifera* (Artz & Nault, 2011; McGrady et al., 2019; Nicodemo et al., 2009; Tepedino, 1981; Walters & Taylor, 2006). In other countries (e.g., Germany and Asian countries), it has been reported that honeybees (*Apis* spp.), bumblebees, and Halictid bees (Halictidae spp.) pollinate, or at least visit the flowers (Ali et al., 2014; Hoehn et al., 2008; Pfister et al., 2017). On the other hand, in Japan, elucidation of pollinator importance of each insect for squashes is scarce, except for a few studies conducted in northern (Hokkaido) and southern (Kagoshima in Kyushu) areas (Abe & Kumano, 2017; Kamo et al., 2022). In other words, we still lack information for Honshu, the main island of Japan. Because the Japanese archipelago is widely spread latitudinally and longitudinally, the entomofauna and squash‐growing season can vary depending on the areas of Japan. Therefore, more examinations in various areas are needed.

The objective of our study was to clarify flower‐visiting insects of *Cucurbita maxima* Duchesne cv. Ebisu (Cucurbitales: Cucurbitaceae) on Honshu and Kyushu, and assess their relative importance as pollinators, considering geographic and diurnal variations. For the frequency of flower visits, we evaluated captured numbers of flower‐visiting insects within a certain time period. Although the definition and measurements of pollination effectiveness are controversial (Gross, 2005; Inouye et al., 1994; Ne'Eman et al., 2010), in this study, we assessed the number of pollen loads of insects and the probability of stigma contact as indices of pollination effectiveness for the following reasons. Our primary objective was to seek flower‐visiting insects which are more likely to contribute to pollination and seed set in the non‐native areas of *C. maxima*, rather than to strictly assess each pollinator's quantitative contribution to seed set. Since squash flowers are self‐compatible and close within a day, invalid pollination due to self or old‐deactivated pollen hardly occurs in the morning when stigmas are receptible (also see the explanation in *Plant*); thus, it is also difficult to suppose that insects, visiting early morning, having abundant squash pollen and touching stigmas transfer entirely no effective pollen or that the composition of invalid pollen highly depends on flower visitors' taxa. Therefore, insects' pollen loads and probability of stigma touching are enough to achieve our aim, and are regarded as a parsimonious approach when one needs to clarify pollinators in various distant sites where even flower visitors of focal plants have been unknown under limited resources. In addition, we examined the body size and behaviors of flower‐visiting insects to aid interpretation of their pollination effectiveness. Finally, by comparing flowers with experimentally manipulated pollination levels and naturally pollinated flowers, we evaluated whether and how much the local insect assemblage provides pollination services for the fruit and seed sets of *C. maxima*.

## 2 MATERIALS AND METHODS

### 2.1 Plant

*Cucurbita maxima* is an annual crop estimated to have originated in South America (Sanjur et al., 2002), and the cultivar Ebisu we are focusing on here is a popular cultivar in Japan. The plant blooms about 2 months after sprouting, and the flowering period of each plant is about a month. Yellow and showy (about 10–20 cm in diameter) flowers bloom at dawn and close about noon on the same day (Ashworth & Galetto, 2001). Although the flower is self‐compatible, pollinating animals are necessary for pollination because the pistillate and staminate flowers bloom separately in the same plant. In addition, the large and sticky pollen grains are unsuited to wind pollination (Bomfim et al., 2016). Previous work in Japan has reported that the abilities of pollen germination and fertilization of squash drastically decrease after anthesis and are almost lost after only several hours (Hayase, 1974b), although high humidity (>70%) may prolong pollen germination ability (Hayase, 1974a). Both pistillate and staminate flowers provide nectar from a base of styles so that insects need to enter deeply into the corolla to get nectar.

### 2.2 Study areas

As study sites, we selected Ibaraki, Mie, and Kagoshima prefecture, because Ibaraki and Kagoshima prefectures are some of the highest production areas of squashes in Japan, and Mie locates geographically intermediately between the two prefectures, longitudinally and latitudinally. Surveys and experiments were conducted at experimental fields of a university and two research institutes: the University of Tsukuba (36.12 N, 140.09E) in Ibaraki prefecture, the Institute of Vegetable and Floriculture Science, NARO (34.77 N, 136.43E) in Mie prefecture, and Kagoshima Prefectural Institute for Agricultural Development (31.48 N, 130.34E) in Kagoshima prefecture. Climate information for those areas is shown in Table A1.

### 2.3 Flower visitor survey

To clarify relative pollinator importance of the flower visitors, considering diurnal and geographic variations, we conducted (1) flower visitor sampling, (2) assessment of the amount of pollen on body surfaces, and (3) the examination of insect behaviors, from May to July 2019. Study plots (25–50 m^2^) were established on experimental fields of *C. maxima* in Ibaraki, Mie, and Kagoshima, and at each plot, insect sampling was conducted for 5 min per hour.

We captured each insect individual on male and female flowers with a 5‐mL plastic vial directly or with an insect net (36 cm in diameter) by sweeping very slowly and then into the vial. Those vials were immediately put into cooler boxes, to keep the insects calm and to not let pollen grains fall. After the survey, all the vials containing insect individuals were labeled and stored in freezers at −20°C. Since the flowering period and the active time of flower visitors depend on daily temperature and sunrise time, the sampling period was optimized at each site. Specifically, collections were conducted at 10 plots from 5:00 to 11:00 on 17 and 27 June, and 9 July in Ibaraki, at three plots from 5:00 to 12:00 on 19 and 25 June and 3 and 5 July in Mie, and at three plots from 6:00 to 11:00 on 21 and 29 May in Kagoshima. Additionally, extra samples from Kagoshima collected on 8 May were added to increase the replications for pollen load assessment. Specimens were morphologically identified to the lowest taxonomic level where possible at the conservation ecology laboratory at the University of Tsukuba. Identification of Halictidae and Nitidulidae (Coleoptera) was helped by expert taxonomists. Bees and large wasps were identified to species, genus, tribe, or family level. Other insect groups were identified to order level at least.

The amount of pollen load of each insect was estimated, following the method described in detail in Nikkeshi et al. (2019). Briefly, we cut and eliminated hind legs with corbicular pollen loads using scissors and poured 0.4 M of sucrose solution (1.0–6.0 mL, depending on the body size) into each vial with an insect sample. This solution is an isotonic solution for pollen (Nikkeshi, 2022) and prevents pollen accumulating at the bottom due to its high viscosity (Nikkeshi, 2022; Nikkeshi et al., 2021). After shaking it in order to separate pollen from the insect bodies and uniformize it in the solution, we sampled 10 μL of the solution, and counted the number of pollen grains on a microscope slide by microscope. In this process, we counted only squash pollen, which has a remarkably larger size (more than 170 μm) compared with the other plant species in our experimental gardens. We repeated this sampling and counting five times, and calculated the average number of pollen grains per 10 μL. Finally, we estimated pollen loads of each insect, the total number of pollen grains adhered to each body surface except for the hind legs, by multiplying the average number of pollen grains per 10 μL and the initial solution volume.

To examine the insects' movement after landing on a flower, we observed female and male flowers for 15 min, and recorded insect behaviors considering four categories (i.e., pollen foraging, nectar foraging, wandering, and unknown). In the case of female flowers, we recorded whether the observed insects touched the stigmas. We defined “pollen foraging” as behaviors that involve mouthparts contacting anthers, or collecting pollen by rubbing the legs. “Nectar foraging” involves visitors inserting their heads or proboscis into the nectary at the flower base, and “wandering” involves walking around or standing still on the flower without foraging. In this observation, *A. mellifera* and *A. cerana japonica* were categorized as “honeybees” due to the difficulty of discriminating between two *Apis* species in the field. This observation was made from 7:00 to 10:30 on 7 days from June 19 to July 8 in Ibaraki (8.25 h in total) and from 7:30 to 11:30 on May 15 and 17 in Kagoshima (2.5 h in total).

### 2.4 Assessment of the contribution of the flower visitors to the squash yield

To verify the smallest body size of flower‐visiting insects that contribute to the fruit and/or seed set, we conducted an enclosure experiment manipulating accessibility to flowers depending on the body size of insects. One day before anthesis, female buds were randomly assigned to enclosure treatments: (1) non‐woven bag (Daiso Industries, CO., Ltd, Hiroshima, Japan), (2) fine mesh bag (inner length: 4.44 × 2.92 mm: “4 mm mesh”, Tomoyasu Works, CO., Ltd., Osaka, Japan), (3) coarse mesh bag (inner length: 7.04 × 7.63 mm: “9 mm mesh”, Tomoyasu Works, CO., Ltd., Osaka, Japan), (4) open pollination, and (5) supplementary hand pollination. Non‐woven bags were supposed to admit no insects, fine mesh bags were supposed to admit Halictidae and other tiny insects, and coarse mesh bags were supposed to admit all insects visiting squash flowers, including honeybees and bumblebees. In addition, by comparing treatments (4) and (5), we examined whether fruit and seed productions were limited by the amount of pollen reception of stigmas in the natural condition. A series of experiments was carried out from June 21 to July 8 in Ibaraki (7 replications), from June 6 to 25 in Mie (14 replications), and on May 16 in Kagoshima (5 replications). To confirm effectiveness of bag control treatment (i.e., coarse mesh bag), we walked around near the experimental flowers, and observed that honeybees and *Bombus diversus diversus* entered the coarse mesh bags.

To protect the experimental flowers from resource competition within the same vine, we eliminated female flowers and buds positioned within five joints from the targeted flowers in Ibaraki, and all female flowers and buds other than the targeted flowers in Mie and Kagoshima. One week after the experimental day, we checked whether the experimental flowers set fruits. After fruit maturation, we compared the number of seeds per fruit and fruit weight between the open (i.e., natural) pollination and the supplementary hand pollination condition.

At a laboratory, to further ensure the accuracy of enclosure treatment, we measured the thorax width of the specimens using a microscope to compare with the inner length of meshes (Sonoda et al., 2022). Since the thorax of Hymenoptera is thicker than the other orders, we also measured the length of the thorax (i.e., the maximum length of the thorax from the back to front). Because the thorax width of Coleoptera tends to be shorter than the abdomen, we measured maximum width of their abdomens. Finally, we tested whether the specimens were able to pass through the mesh physically by hand, and confirmed our experimental setting worked as intended (Table A2).

### 2.5 Statistical analyses

All analyses were performed using R 4.2.0 software (R CoreTeam, 2022).

#### 2.5.1 Insect behavior on flowers

Before the analyses, data for aphids, caterpillars, and ants were excepted because of no replicative observation (*N* = 1). We tested whether the probability of contacting stigmas of the each flower‐visiting insect depended on the insect group (*B. d. diversus*, honeybees, halictid bees, thrips, small beetles, and flies) and/or areas (Ibaraki and Kagoshima) as main effects. This analysis was performed by a generalized linear model (GLM), assuming the binomial error with logit link function, using the glmmTMB function of “glmmTMB” package (Brooks et al., 2020). Significance of the experimental variables was tested by likelihood ratio test (function “lrtest”) and then post hoc tests (Tukey–Kramer) were conducted to assess the differences among insect groups.

We also analyzed the proportion of the categorized insect behavior after landing on flowers to examine the insect habits which potentially influence pollination effectiveness. The proportion of categories of insect behaviors on a flower was analyzed by a multinomial logistic regression model, using the function “vglm” of package ‘VGAM’ (Yee, 2015). In this model, a multinomial variable with three values (pollen foraging, nectar foraging, and wandering) was the response variable, and insect groups (*B. d. diversus*, honeybees, halictid bees, thrips, small beetles, and flies) and areas (Ibaraki and Kagoshima) were the explanatory variables. Significance of explanatory variables was assessed by likelihood ratio test (function “lrtest”). As post hoc tests, pairwise comparisons among insect groups were carried out in the same sex of flowers, applying the Bonferroni correction (adjusted the *p*‐value at .05/15).

#### 2.5.2 Geographic and diurnal variation of bee abundance, pollen loads, and stigma contact

Since *B. d. diversus*, *A. mellifera*, *A. c. japonica*, and Halictidae were the major flower visitors with abundant pollen loads, we tried to phenomenologically understand how (1) the captured number and (2) pollen loads of those bees change depending on the time elapsed within a day, and whether and how the patterns can be varied among the study areas and flower sex. To express those patterns by the best approximate model, we followed the statistical procedure recommended by Zuur et al. (2009). This procedure involved constructing a model which contains as many explanatory variables and their interactions as possible, visually checking validation of homogeneity of the residuals, selecting the appropriate random structure, and then selecting the appropriate fixed variables based on model indices. Specifically, we performed GLMs and generalized linear mixed models (GLMMs) by “glmmTMB” package (Brooks et al., 2020). In the models, flower sex, study area, time point, squared time point, and those quadratic interactions were set as explanatory variables. As an exception, we did not include study area for the models of *B. d. diversus*, since this species was collected only in Ibaraki. The date of the survey and the identity of experimental plots were set as random intercepts if it improved homogeneity of the residuals. We assumed an appropriate variance–covariance structure (AR (1) or compound symmetry), if temporal autocorrelation was detected and was weakened by addition of the structures. For the captured number and pollen loads of bees, which are count data, we either assumed Poisson or Negative binomial with log link function, depending on the results of model validation. The error distribution and random terms for the final models are shown in Table A3. Selection of the fixed effects was based on AICc (function “dredge”: MuMIn). We regarded the models with delta AICc <2.0 as plausible. The best model (delta AICc = 0.0) was used for visualization using the package “ggeffects”. In the only case of pollen loads of *B. d. diversus*, the fourth model (delta AICc = 0.45) was selected for visual expression because the other selected models included only single factors and were thus unable to show the relationships between flower sex and time. Validation for the best model was conducted by the function “simulateResiduals” of package “DHARMa” (Hartig, 2022).

Regarding the probability of stigma contact of the bees, we set the presence or absence of stigma contact per visit as a response variable (binomial error distribution with logit link function). The timing of visits, taxa, and their interactions were set as explanatory variables. Areas and trial IDs were included as random effects for model convergence. Model selection and validation were conducted following the same procedure as above.

#### 2.5.3 Fruit and seed set

To evaluate pollination deficits in the natural condition, we analyzed the differences in reproductive parameters (fruit set, the number of seeds, and fruit weight) of *C. maxima* between the conditions of natural and supplementary hand pollination. For the analyses of the fruit set, fruit weight, and the number of seeds, area (Ibaraki, Mie, and Kagoshima) and their interactions with flower treatment were added as explanatory variables, and the flowering day was included as a random term. For fruit weight as continuous data and the number of seeds as count data, Gaussian error distribution and negative binomial error distribution with log link function were used, respectively. These analyses were performed by the package “glmmTMB.”

## 3 RESULTS

We collected 781 flower‐visiting individuals in Ibaraki, Mie, and Kagoshima in the regular surveys (Table 1). The collection consisted of *A. mellifera* (50.7%), *A. cerana* (25.6%), *B. d. diversus* (11.7%), Halictidae (5.0%, including *Lasioglossum* and *Halictus*), and other taxonomic groups (<2.0%). Those bees tended to have abundant pollen grains on their body surfaces (*A. mellifera*: 297 ± 310; *A. cerana*: 179 ± 266; *B. d. diversus*: 1870 ± 1168; Halictidae: 624 ± 1504; mean ± SD were shown). In the minor groups, the following taxa also had relatively high pollen loads (more than 100 pollen grains on average): *Bombus ignitus* (1528, from one individual), Campsomerini (452 ± 456), *Eucera spurcatipes* (180, from one individual), and *Xylocopa tranquebarorum tranquebarorum* (118 ± 84).

**TABLE 1:** A list of flower‐visiting insects of *Cucurbita maxima* at the three study sites, and the number of pollen grains adhered to each body surface.

Area	Order	Family	Species	Flower sex	No. of individuals captured	No. of individuals for pollen analysis	Pollen grains	
							Mean	SD
Ibaraki	Hymenoptera	Apidae	*Bombus diversus diversus*	F	26	26	2397	1303
			*Bombus diversus diversus*	M	65	25	1322	654
			*Apis mellifera*	F	7	7	170	80
			*Apis mellifera*	M	83	26	258	153
			*Apis cerana japonica*	M	1	0	–	–
		Halictidae	Halictidae spp.	F	3	3	83	74
			Halictidae spp.	M	8	8	1692	2412
		Scoliidae	Campsomerini spp.	M	2	2	1004	572
		Unidentified	Unidentified wasp	M	1	1	24	–
	Coleoptera	Nitidulidae	Nitidulidae sp.	F	1	1	3	–
			Nitidulidae spp.	M	11	11	12	11
		Scarabaeidae	Cetoniinae spp.	M	2	2	102	74
		Chrysomelidae	*Aulacophora nigripennis*	M	6	6	3	5
			*Aulacophora femoralis*	M	3	3	5	7
		Unidentified	Unidentified Coleoptera	M	1	1	0	
	Diptera	Unidentified	Unidentified Diptera	M	1	1	26	–
	Hemiptera	Unidentified	Unidentified Hemiptera	M	1	1	0	–
	Lepidoptera	Unidentified	Unidentified caterpillar	F	1	0	–	–
			Unidentified caterpillars	M	8	0	–	–
Mie	Hymenoptera	Apidae	*Apis mellifera*	F	40	25	144	188
			*Apis mellifera*	M	120	27	413	356
			*Apis cerana japonica*	F	127	25	96	154
			*Apis cerana japonica*	M	68	23	287	313
			*Eucera spurcatipes*	M	1	1	180	–
			*Xylocopa tranquebarorum tranquebarorum*	F	2	2	128	16
			*Xylocopa tranquebarorum tranquebarorum*	M	3	3	112	108
		Halictidae	Halictidae spp.	F	3	3	8	11
			Halictidae spp.	M	20	9	31	41
		Scoliidae	*Campsomeris annulata*	F	1	1	416	–
			*Campsomeris annulata*	M	3	3	241	199
			*Megacampsomeris prismatica*	M	1	1	76	–
	Coleoptera	Scarabaeidae	*Oxycetonia jucunda*	M	1	1	20	–
Kagoshima	Hymenoptera	Apidae	*Bombus ignitus*	M	0	1^a^	1528	–
			*Apis mellifera*	F	72	25	204	295
			*Apis mellifera*	M	74	25	493	379
			*Apis cerana japonica*	F	1	8^a^	11	10
			*Apis cerana japonica*	M	3	6^a^	339	348
		Halictidae	Halictidae spp.	F	1	2^a^	18	24
			Halictidae spp.	M	4	5^a^	919	1084
		Scoliidae	*Campsomeris annulata*	F	0	1^a^	388	–
		Unidentified	Unidentified micro wasp	M	1	0	–	–
	Coleoptera	Chrysomeridae	Chrysomeridae sp.	M	1	0	–	–
	Diptera	Unidentified	Unidentified Diptera	F	2	1	0	–
			Unidentified Diptera	M	1	1	56	–

^a^ Additional samples were collected for pollen analysis, and thus the number of individuals for pollen analysis exceeded that of captured.

By region, we collected 231 individuals. *B. d. diversus* (39.4%) and *A. mellifera* (39.0%) made up more than three‐quarters of flower visitors in Ibaraki. This was followed by Nitidulidae (5.2%) and Halictidae (4.8%). In Mie, we collected 390 individuals, and the percentages of *A. mellifera* and *A. c. japonica* were almost equally high (i.e., 41.0% and 50.0%, respectively), followed by Halictidae (5.9%). In Kagoshima, 160 individuals were collected, and *A. mellifera*, *A. c. japonica*, and Halictidae composed 90.0%, 2.5%, and 3.1%, respectively.

**FIGURE 1:**
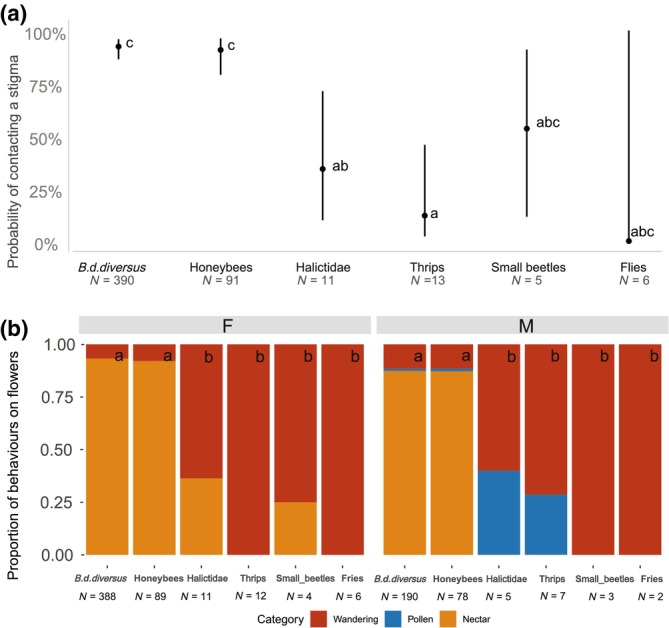
The probability of contacting stigmas (a) and the proportion of insect behavior on a flower, including both male and female flowers (b). “F” and “M” indicates female and male flowers, respectively. “*N*” represents the number of observations. Values accompanied by the same letters above the point or on the bars indicate not differing significantly among taxa.

The probability of stigma contacts was significantly influenced by insect taxa but not area (taxa: df = 5, *χ* ^2^ = 73.1, *p* < .0001; area: df = 1, *χ* ^2^ = 0.1, *p* = .7239), and *B. d. diversus* and honeybees showed significantly higher probability of touching than Halictidae and thrips (Figure 1a). The proportions of each behavior after landing on a flower differed among insect taxa (df = 10, *χ* ^2^ = 174.0, *p* < .0001) and floral sex (df = 4, *χ* ^2^ = 22.6, *p* = .0002) significantly, whereas area had weak effects on it (df = 2, *χ* ^2^ = 5.1, *p* = .079). Specifically, *B. d. diversus* and honeybees mainly foraged on nectar on the flowers, and the proportions of behaviors were significantly different from the other taxa, on both the male and female flowers (Figure 1b).

**FIGURE 2:**
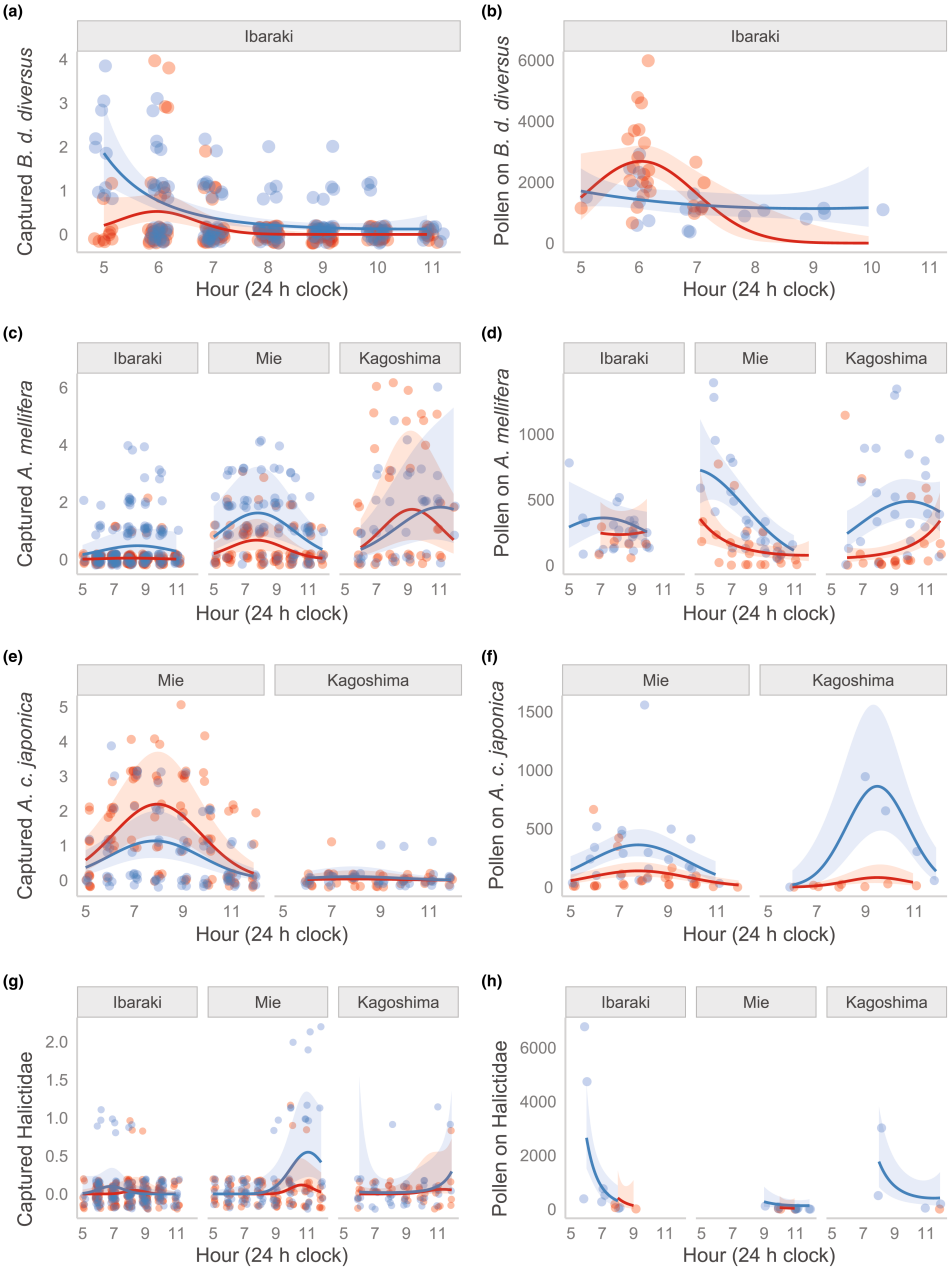
Diurnal variations in the captured number (a, c, e, g) and the estimated number of pollen grains on the bodies (b, d, f, h) of flower‐visiting bees (*Bombus diversus diversus*, *Apis mellifera*, *Apis cerana japonica*, and Halictidae) of *Cucurbita maxima* at three sites. Colors of circles indicate flower sex at which the focal insects were captured (blue: male; red: female). Predicted relationships between time and number of captured insects per time, and those 95% CIs are shown by lines and shades, respectively. The time range from 5:00 to 11:00 (a and b) and 5:00 to 12:00 (c–h) are shown. Note that the survey durations depend on the sampling areas and dates (see Section 2). Time periods with no data are shown as blank.

Flower sex, time, and area influenced the numbers of the four major bees and their pollen loads in bee species‐specific manners (Figure 2, Tables A4 and A5). In the case of *B. d. diversus* which was only observed in Ibaraki, the numbers decreased as time passed, though the peak time was slightly different between flower sexes (Figure 2a, Table A4: flower sex × time, flower sex × time^2^). In synchrony with the visits, the pollen loads of *B. d. diversus* decreased as time passed, depending on flower sex (Figure 2b, Table A5: flower sex × time, flower sex × time^2^). The captured number of *A. mellifera* tended to be high around 8:00 to 9:00 (Figure 2c), but highly dependent on area and flower sex (Table A4: interaction terms among time, time^2^, area, and flower sex). Temporal dynamics of their pollen loads were also variable depending on area and flower sex (Table A5: main effects and interactions), and its tendency was not synchronized with the captured number (Figure 2c,d). The number of *A. c. japonica* was high around 8:00 in both areas (Figure 2e, Table A4: main effects of time and time^2^), while it was consistently low in Kagoshima. The pollen loads in Mie showed a similar pattern to the captured number, but the peak of pollen loads in Kagoshima was at around 9:00 (Figure 2f, Table A5: interaction between time^2^ and area). The number of Halictidae peaked around 11:00, except for in Ibaraki (Figure 2g, Table A4: interaction between time^2^ and area), whereas the pollen loads decreased as time passed in all the areas (Figure 2h, Table A5: no interaction terms between area and time).

**FIGURE 3:**
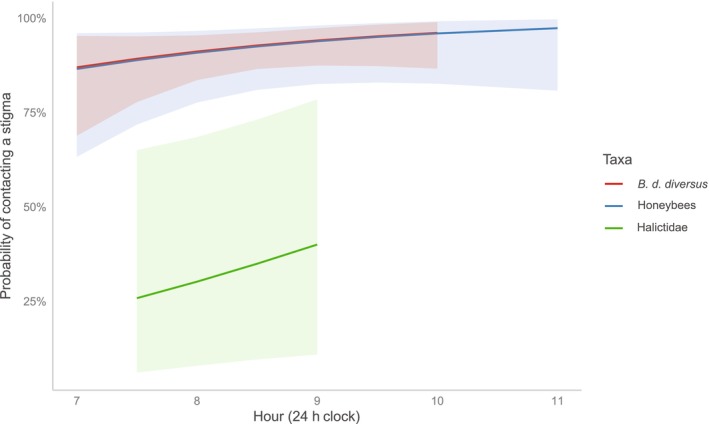
Temporal changes in the probability of contacting stigma of *Bombus diversus diversus*, honeybees, and Halictidae. Predicted relationships between time and the probability of contacting stigma, and those 95% CIs are shown by lines and shades, respectively.

As a result of the model selection approach, it was clarified that the best approximate model (delta AICc = 0.00) for the probability of stigma contact contained taxa as a fixed effect, and the second model (delta AICc = 0.39) contained both time and taxa. The probabilities of stigma contact gradually increased with time, and those of *B. d. diversus* and honeybees tended to be higher than Halictidae constantly (Figure 3).

**FIGURE 4:**
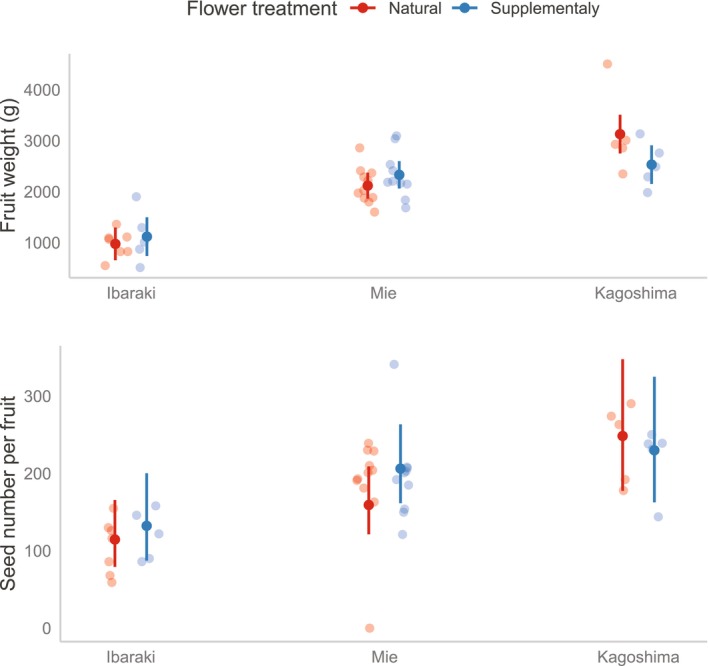
Fruit weight (top) and seed number per fruit (bottom) in the condition of natural and supplementary hand pollination in Ibaraki, Mie, and Kagoshima. Closed circles and vertical bars represent marginal means and 95% CIs, respectively. Translucent circles show raw data. No significant differences were detected between the flower treatments at all the sites.

Flowers covered with coarse (approximately 9 mm) mesh bags set fruits, except in Kagoshima (Table A6). Supplementary hand pollination did not increase the proportion of fruit set in 2019 (Figure 4: treatment: df = 1, *χ* ^2^ = 1.85, *p* = .1739, Table A5), irrespective of the area (area: df = 2, χ^2^ = 0.57, *p* = .753; area × treatment: df = 2, χ^2^ = 1.69, *p* = .429). Furthermore, fruit weight was only affected by area (df = 2, *χ* ^2^ = 23.7, *p* < .0001), and was not affected by treatments throughout the three areas (Figure 4; treatment: df = 1, *χ* ^2^ = 0.34, *p* = .560; treatment × area: df = 2, *χ* ^2^ = 5.93, *p* = .052). This tendency was also seen in the number of seeds per fruit (Figure 4; treatment: df = 1, *χ* ^2^ = 0.68, *p* = .410; area: df = 2, *χ* ^2^ = 7.45, *p* = .024; treatment × area: df = 2, *χ* ^2^ = 1.44, *p* = .487).

## 4 DISCUSSION

*Cucurbita maxima* were mainly visited by the bees with abundant pollen and often touching stigma, including *A. mellifera*, *A. c. japonica*, Halictidae, and *Bombus d. diversus* at our study sites, whereas species composition differed among the sites. Although the other Apocrita (Hymenoptera) also had pollen loads as high as the major bees, their intensities of flower visits were scarce in the areas under study. Therefore, their pollinator importance as a product of the intensity and effectiveness should be low in the three sites at present. Nitidulidae visited flowers relatively frequently in Ibaraki, but this beetle had only 12 pollen grains on average. Thus, it seems that Nitidulidae could deposit only a few pollen grains of *C. maxima* per visit, even if they touched a stigma once every two visits. From here, we mainly focus on discussing the above four major bees as important pollinators in the studied areas.

### 4.1 Geographic variations of pollinators and reproductive success of *C. maxima*

This study clearly demonstrated geographic variations of pollinators of *C. maxima* in Japan. At our study sites, *A. mellifera*, visited flowers of *C. maxima*, with different dominant species at different sites. We did not introduce hives of *A. mellifera* in this experiment, but they were observed at all three sites. This implies that introduced *A. mellifera* for honey or crop pollination plays a role as a pollinator for *C. maxima*, although this was not originally intended by beekeepers. On the other hand, although all the sites were in the natural distribution range of *B. d. diversus* (Kinota et al., 2013), this species was only observed in Ibaraki. This result supported our preliminary survey of *C. maxima* conducted in the same sites and season from 2017 to 2018 (Ikemoto unpublished data and Ikemoto et al., 2021). Although the previous study carried out at the same site of Kagoshima in autumn 2019 and 2020 (Kamo et al., 2022) reported *B. d. diversus* visiting *C. maxima*, we did not find any individuals in this study. Such an inconsistent result between the two studies could simply be because the experimental periods in our study were earlier than the appearance season of the workers (Kinota et al., 2013). Although the reason why *B. d. diversus* were not observed in Mie is unknown, a recent study reported that the estimated range of *B. diversus* has shrunk in Japan probably due to forest loss (Suzuki‐Ohno et al., 2020). Therefore, such a recent threat to *B. diversus* may have potentially influenced our results, although further studies are needed to confirm this.

Production of fruits and seeds was not limited by natural pollination in all areas (Figure 4), suggesting that insect pollinators provide sufficient pollination services for *C. maxima*, as with previous squash studies conducted in other areas (McGrady et al., 2019; Pfister et al., 2017), despite geographic variations of species composition. It should be carefully considered that visits by *A. mellifera* totally depend on the circumstances of beekeepers, including occurrence of honeybee disease and availability of commercial hives. Therefore, maintaining wild bee populations is likely necessary for the sustainable pollination success of *C. maxima*.

### 4.2 Diurnal variations of pollinator importance of each bee

To our knowledge, this is a rare study showing diurnal variations of pollination effectiveness within a taxon (but see Pfister et al., 2017). Interestingly, temporal dynamics of pollen loads and probability of stigma contacts depended on taxa, and did not necessarily synchronize with the dynamics of flower visits (i.e., captured number of individuals). In particular, the visits and probability of stigma contact of Halictidae tended to increase as time passed, in contrast to their pollen loads that sharply decreased as time passed. Therefore, the contribution of most Halictidae individuals to pollination would be low. In fact, the flowers with fine mesh which Halictidae could enter (Table A2) did not set fruit at any of the sites (Table A6). Pfister et al. (2017) indicated that Halictidae is a pollinator for *C. maxima* cv. Hokkaido, but its efficiency is low because (1) it is relatively less hairy and so cannot carry much pollen, and because of (2) the low probability of touching the reproductive parts of squash flowers due to their small bodies. Our study partly supported explanation (2), as shown in Figures 1 and 3, but did not support explanation (1); the range of pollen loads of Halictidae was even higher than that of honeybees, especially in the early morning (Figure 2). This could be due to their pollen‐foraging behavior (Figure 1b). Thus, the pollinator importance of Halictidae seemed to be relatively low in our present study, mainly due to their unsynchronized timing of maximum pollen loads and visitation frequency, and little stigma touching.

In contrast, temporal dynamics of the number of flower visits and pollen loads of *B. d. diversus* were well synchronized, and the probability of stigma contact remained high through time (Figure 3). The decrease in the pollen loads of *B. d. diversus* supports a previous report on *C. maxima* that the amount of pollen deposition on a stigma by bumblebees decreases with time (Pfister et al., 2017). All findings in this study suggest that *B. d. diversus* are the most effective pollinators, as with the previous studies reporting that bumblebees are effective in other countries and areas (see references in the introduction).

Regarding the two related *Apis* species, pollen loads of *A. c. japonica* were highly synchronized with the visits, especially in Mie. In contrast, those of *A. mellifera* varied among the areas, and tended to be low during the peak times of their visits. Many studies have reported that *A. mellifera* is an important pollinator of squash since their frequent flower visits often compensate for the disadvantage of low pollination efficiencies per visit (Artz & Nault, 2011; Kamo et al., 2022; Pfister et al., 2017; Walters & Taylor, 2006). On the other hand, there are few reports of *A. cerana* as pollinators for squash (but see Hoehn et al., 2008). Considering the synchronization of peak timing of their pollen loads and flower visits, *A. c. japonica* can be an even better squash pollinator than *A. mellifera*, although further studies are needed to examine the exact difference in pollination abilities (e.g., amount of stigmatic pollen per visit) between the two honeybee species.

### 4.3 Implications for sustainable agriculture management

With the recent global need for the monitoring and elucidation of crop pollinators, simple and efficient monitoring methods are required. For example, there are challenges in evaluating pollination deficits of commercial crops, by examining whether the total number of flower visits exceeds pollination thresholds estimated from information on the amount of deposited pollen on a stigma per visit and the least amount of pollen for fruit (e.g., Garibaldi et al., 2020; McGrady et al., 2019). Such process‐based approaches are important to calculate realistic economic value that the natural/managed pollinators at the sites produce. Estimation of stigmatic pollen amount per visit may need a careful survey, because it may vary over only a few hours, as suggested by Pfister et al., 2017 directly, and by this study indirectly. On the other hand, there are numerous crop fields where even flower visitors are unknown. In such situations, as a first step, pattern‐based studies that ensure (1) which insects frequently visit the flowers and potentially deposit pollen, (2) what kinds of behavioral and morphological traits important pollinators have, and (3) whether those pollinator assemblages provide sufficient pollen for fruit production will be also helpful to fill the knowledge gap of pollination by natural insects.

As global warming progresses, time suitable for bee activity will become shortened within a day in the summer, which may change plant–pollinator relationships. In the case of *C. maxima*, the activity time of bumblebees may become earlier and thus they would visit fewer flowers, because their flight performance is restricted in high temperatures (Kenna et al., 2021). Pollinator importance of Halictidae for *C. maxima* may also be strongly influenced by global warming; they may come to pollinate more, if their visits occur earlier when more individuals keep high pollen loads. We should also pay attention to the future dynamics of minor flower visitors that may become important pollinators even if intensity of floral visits increases. Taken together, even though our study does not show the diversity‐function relationships of pollinators and fruit production directly, it indirectly suggests the importance of maintaining wild bee diversity for agricultural sustainability, as insurance against a fluctuating environment (Yachi & Loreau, 1999).

## AUTHOR CONTRIBUTIONS

**Mito Ikemoto:** Conceptualization (lead); data curation (lead); formal analysis (lead); investigation (lead); methodology (lead); project administration (supporting); resources (equal); validation (lead); writing – original draft (lead). **Yoshihiro Tanaka:** Data curation (supporting); investigation (equal); methodology (equal); resources (equal); writing – original draft (supporting). **Katsuyuki Kohno:** Data curation (supporting); investigation (equal); methodology (supporting); resources (equal); writing – original draft (supporting). **Tomoyuki Yokoi:** Conceptualization (supporting); funding acquisition (lead); investigation (supporting); methodology (supporting); project administration (lead); supervision (lead); writing – original draft (supporting).

## CONFLICT OF INTEREST STATEMENT

The authors declare no conflicts of interest.

## Data Availability

Data are available at Dryad Digital Repository (DOI: 10.5061/dryad.vmcvdnczm).

## APPENDIX 1

**TABLE A1:** Monthly weather information during the survey in 2019 obtained from the nearest weather observation station of Japan Meteorological Agency to each experiment field.

Observation station of JMA	Longitude	Latitude	Month	Total precipitation per month (mm)	Temperature (°C)			Humidity	
					Average (°C)	Maximum (°C)	Minimum (°C)	Average (%)	Minimum (%)
Tsukuba (Ibaraki)	N36°03.4′	E140°07.5′	June	141.5	20.9	30.6	13.4	82	34
			July	160	23.5	34.2	17.3	88	51
Tsu (Mie)	N34°44′	E136°31.1′	June	191	22.8	32.7	17.2	69	21
			July	342.5	25.5	34.5	20.1	77	47
Kaseda (Kagoshima)	N31°24.9′	E130°19.5′	May	99.5	20.1	30.3	9.9	‐	‐

**TABLE A2:** The body size of insects in taxonomic group, and the percentage of specimens passing through the nets of each mesh size (fine: 4 mm; coarse: 9 mm).

Order	Species	Area	*N*	Thorax or abdomen width (mm)		Thorax height (mm)		Percentage for coarse mesh	Percentage for fine mesh
				Mean	SD	Mean	SD		
Hymenoptera	*Bombus diversus diversus*	Ibaraki	8	5.80	0.33	6.34	0.42	1.00	0.00
	*Bombus ignitus*	Kagoshima	1	8.23	–	7.98	–	0.00	0.00
	*Apis mellifera*	Ibaraki	6	3.99	0.19	3.76	0.19	1.00	0.00
		Mie	6	3.97	0.20	3.86	0.15	1.00	0.00
		Kagoshima	6	3.85	0.25	3.63	0.20	0.83	0.00
	*Apis cerana japonica*	Mie	6	3.59	0.10	3.40	0.54	1.00	0.00
		Kagoshima	6	3.91	0.21	4.10	0.24	1.00	0.00
	*Xylocopa (Biluna) tranquebarorum tranquebarorum*	Mie	5	6.86	0.76	7.52	0.77	0.60	0.00
	*Eucera spurcatipes*	Mie	1	4.18	–	3.54	–	1.00	0.00
	Halictidae spp.	Ibaraki	8	2.40	0.39	2.29	0.35	1.00	1.00
		Mie	7	2.44	0.65	2.39	0.67	1.00	0.86
		Kagoshima	7	2.67	0.77	2.45	0.65	1.00	0.86
	Campsomerini spp.	Ibaraki	2	4.26	0.24	4.43	0.43	1.00	0.00
		Mie	5	4.34	0.24	4.52	0.46	1.00	0.00
		Kagoshima	2	4.80	0.09	5.06	0.37	1.00	0.00
Diptera	Diptera spp.	Kagoshima	2	1.73	0.63	–	–	1.00	1.00
	Syrphidae spp.	Kagoshima	2	1.53	0.01	–	–	1.00	1.00
Coleoptera	Coleoptera spp.	Ibaraki	8	2.26	1.30	–	–	1.00	1.00
	*Aulacophora* sp.	Kagoshima	1	3.83	–	–	–	1.00	1.00
	Cetoniinae spp.	Ibaraki	2	7.44	0.31	–	–	1.00	0.00
		Mie	1	6.97	–	–	–	1.00	0.00

**TABLE A3:** Error distributions and random terms for each model.

Attribute	Taxa	Error distribution	Random term
Captured number	*Bombus diversus diversus*	Negative binomial	–
	*Apis mellifera*	Negative binomial	Time:AR (1)
	*Apis cerana japonica*	Poisson	Time:AR (1)
	Halictidae	Poisson	Day
Pollen loads	*Bombus diversus diversus*	Negative binomial	Plot
	*Apis mellifera*	Negative binomial	–
	*Apis cerana japonica*	Negative binomial	–
	Halictidae	Negative binomial	Plot

**TABLE A4:** Selected top models for the captured number of *Bombus diversus diversus*, *Apis mellifera*, *Apis cerana japonica*, and Halictidae.

Taxon	Model rank	Area	Flower sex	Time	Time^2^	Area × flower sex	Area × time	Area × time^2^	Flower sex × time	Flower sex × time^2^	df	AICc	Δ AICc
*B. d. diversus*	1		+	11.90	−1.00				+	+	7	355.79	0.00
*A. mellifera*	1	+	+	2.50	−0.15	+		+	+	+	15	1004.43	0.00
	2	+	+	2.45	−0.15	+	+		+	+	15	1005.06	0.64
	3	+	+	1.70	−0.11	+		+		+	14	1005.90	1.48
	4	+	+	1.68	−0.11	+	+			+	14	1006.31	1.89
*A. c. japonica*	1	+	+	2.35	−0.15	+					8	498.31	0.00
	2	+	+	2.83	−0.15	+	+				9	498.71	0.40
	3	+	+	2.39	−0.13	+		+			9	498.78	0.46
	4	+	+	2.34	−0.15						7	498.81	0.50
	5	+	+	2.83	−0.15		+				8	499.19	0.88
	6	+	+	2.39	−0.13			+			8	499.26	0.95
Halictidae	1	+	+	22.63	−1.36	+	+	+	+	+	15	234.11	0.00
	2	+	+	6.34	−0.44		+	+			11	234.70	0.59
	3	+	+	5.88	−0.41			+			9	234.83	0.72
	4	+	+	15.43	−0.92	+		+	+	+	13	235.23	1.12
	5	+	+	11.80	−0.75		+	+	+	+	13	235.32	1.21
	6	+	+	4.23	−0.30		+				9	235.46	1.35
	7	+	+	10.77	−0.68			+	+	+	11	235.57	1.46

*Note*: The models were ranked by AICc. 3rd to 11th columns indicate the candidate variables in the models, and “plus” symbols indicate the selected variables in the corresponding model. Since *Bombus d. diversus* was collected from only Ibaraki, the variable "area" and its interaction terms were not included in the full model (we represented the cells in black shades).

**TABLE A5:** Selected top models for pollen loads of *Bombus diversus diversus*, *Apis mellifera*, *Apis cerana japonica*, and Halictidae.

Taxon	Model rank	Area	Flower sex	Time	Time^2^	Area × flower sex	Area × time	Area × time^2^	Flower sex × time	Flower sex × time^2^	d f	AICc	Δ AICc
*B. d. diversus*	1		+								4	842.16	0
	2		+		−0.01						5	842.33	0.17
	3		+	−0.1							5	842.34	0.19
	4		+	6.58	−0.55				+	+	8	842.6	0.45
*A. mellifera*	1	+	+	−0.55	0.03	+	+		+	+	13	1758.18	0
	2	+	+	−0.05			+				8	1758.83	0.65
	3	+	+	0.05			+		+		9	1759.24	1.06
	4	+	+		0.01	+		+		+	11	1759.4	1.22
	5	+	+	0.06		+	+		+		11	1759.52	1.34
	6	+	+		0			+		+	9	1759.54	1.36
	7	+	+	−0.06		+	+				10	1759.76	1.58
	8	+	+	0.34	−0.02	+	+			+	12	1759.79	1.61
	9	+	+	−0.61	0.04	+		+	+	+	13	1759.95	1.77
	10	+	+		0			+			8	1760.12	1.94
*A. c. japonica*	1	+	+	1.86	−0.12	+	+	+			9	710.11	0
Halictidae	1	+	+	−6.2	0.24				+		9	355.59	0
	2	+	+	−5.23	0.2					+	9	355.75	0.16

*Note*: The models were ranked by AICc. 3rd to 11th columns indicate the candidate variables in the models, and “plus” symbols indicate the selected variables in the corresponding model. Since *Bombus d. diversus* was collected from only Ibaraki, the variable "area" and its interaction terms were not included in the full model (we represented the cells in black shades).

**TABLE A6:** The percentage of fruit set per treatment when enclosed by non‐woven bags, fine mesh bags (approximately 4 mm), coarse mesh bags (approximately 9 mm), open (i.e., natural) or supplementary hand pollination, and the number of replicates per treatment.

	Replicates per treatment	Non‐woven	Fine mesh	Coarse mesh	Open	Supplementary hand pollination
Ibaraki	7	0	0	57.1%	100.0%	71.4%
Mie	14	0	0	28.6%	78.6%	33.3%
Kagoshima	5	0	0	0.0%	100.0%	100.0%
